# Mechanistic Insights into Anti-Nectin4-VcMMAE-Induced Ocular Toxicity: From Cellular Uptake Pathways to Molecular Modification

**DOI:** 10.3390/ijms26114996

**Published:** 2025-05-22

**Authors:** Jialing Zhang, Meng Li, Weiyu Li, Yuxuan Yang, Gang Wu, Peng Guo, Chuanfei Yu, Lan Wang

**Affiliations:** 1 Division of Monoclonal Antibodies, Institute for Biological Product Control, National Institutes for Food and Drug Control, Beijing 102629, China; zhangjialing@nifdc.org.cn (J.Z.); limeng@nifdc.org.cn (M.L.); liweiyu1219@163.com (W.L.); wugang@nifdc.org.cn (G.W.); 2 Hangzhou Institute of Medicine (HIM), Chinese Academy of Sciences, Hangzhou 311121, China; yxyang999@gmail.com (Y.Y.); guopeng@ucas.ac.cn (P.G.); 3 State Key Laboratory of Drug Regulatory Science, National Institutes for Food and Drug Control, Beijing 102629, China

**Keywords:** enfortumab vedotin, ocular toxicity, endocytosis mechanism, molecular optimization

## Abstract

Antibody–drug conjugates (ADCs) represent a novel approach to cancer treatment. Enfortumab vedotin (PADCEV), as a prominent example, has demonstrated remarkable clinical efficacy. However, its ocular toxicity has raised concerns. This study aimed to explore the molecular mechanisms underlying PADCEV—induced ocular toxicity. SD rats, whose ocular structures are similar to those of humans, were selected to establish an ocular toxicity model to mimic the human response. In vitro experiments were conducted using human primary corneal epithelial cells, HCE-T. The results confirmed that nectin-4 plays a crucial role in the cellular uptake of PADCEV, and non-specific pinocytosis is also involved. Additionally, a variant was obtained by introducing point mutations in the Fc region of PADCEV, which was found to reduce corneal epithelial toxicity. The findings of this study not only deepen our understanding of ADC-induced ocular toxicity but also provide new insights into optimizing ADC design and enhancing treatment safety.

## 1. Introduction

Antibody–drug conjugates (ADCs) are novel cancer treatments that combine the specificity of monoclonal antibodies and the efficacy of cytotoxic drugs [1,2,3]. ADCs target specific antigens on the surface of cancer cells via monoclonal antibodies and transport the linked cytotoxin to the intracellular space in targeted cells to selectively kill tumor cells while minimizing damage to normal tissues [4,5,6]. Recent advances in biotechnology and in-depth clinical research contributed to the development of ADCs with significant efficacy as anti-tumor immunological therapies [7,8]. However, ADC therapy has limitations, including ocular toxicity, particularly in patients who receive long-term or multiple drug administrations [9,10]. Among the 15 commercially available ADCs, five drugs cause ocular toxicity, characterized by corneal epithelial damage, conjunctivitis, and dry eye syndrome [11,12]. Ocular toxicity severely affects the quality of life in patients and may lead to vision impairment [13]. The mechanisms of ADC-induced ocular toxicity are poorly understood. Most studies concerning ADC-induced ocular toxicity are limited to the discovery and characterization of clinical cases. Understanding the mechanisms of ADC-induced ocular toxicity is essential for optimizing therapeutic protocols and improving patient safety and tolerance.

Enfortumab vedotin, an anti-Nectin4-VcMMAE (MMAE, monomethyl auristatin E) drug to treat urothelial cancer, obtained accelerated approval from the U.S. Food and Drug Administration in December 2019 [14,15]. Enfortumab, a human IgG1 monoclonal antibody targeting Nectin-4, and monomethyl auristatin E (MMAE), a microtubule inhibitor, are conjugated via a cleavable linker to form anti-Nectin4-VcMMAE. Anti-Nectin4-VcMMAE (enfortumab vedotin) is the first approved programmed cell death protein 1 (PD-1) + ADC combination drug and has a unique competitive advantage in treating urothelial cancer, offering new therapeutic options for patients [15,16]. Since approval and marketing, the sales revenue of anti-Nectin4-VcMMAE increased quickly, reaching 1.03 billion US dollars. Despite the significant clinical success of enfortumab vedotin, the potential ocular toxicity limits its use. Warnings and precautions on the drug label stress that using anti-Nectin4-VcMMAE may cause ocular diseases. Most ocular symptoms occur in the cornea, manifesting as dry eye syndrome and related symptoms. In a study of 310 patients receiving anti-Nectin4-VcMMAE therapy, 46% developed ocular diseases. Adverse effects in the cornea include keratitis, blurred vision, limbal stem cell deficiency, and other dry eye-related events [17].

Human Nectin-4 is 94% homologous to *Macaca fascicularis* and rats. Interestingly, the binding affinity of ADC for Nectin-4 is similar in humans, *Macaca fascicularis*, and rats. Thus, these animal models are suitable for toxicological studies [18]. Pre-clinical 4-week repeated dose toxicity studies in rats and *Macaca fascicularis* and 13-week studies in rats demonstrated that the antibodies alone did not cause ocular diseases. Thus, the ocular toxicity is related to the whole ADC [18].

The human eye is a complex organ with six corneal layers. The surrounding tissue and limbus of the cornea are filled with immunoglobulins, complements, and diverse components for cellular immunity [19]. Therefore, the eye is an immune-privileged tissue, and large molecules have limited access. Only small molecules with specific physicochemical properties, such as monomethyl auristatin F and maytansinoid DM4, can enter the eye. The blood–aqueous barrier inhibits the entrance of ADCs into the aqueous humor, reducing the risk of cornea exposure to ADCs via the front aqueous humor. Furthermore, pre-clinical and clinical studies showed that ADC-related corneal damage is limited to the epithelial layer [20,21]. Anti-Nectin4-VcMMAE is linked via valine-citrulline-p-aminobenzyl carbamate (Val-Cit-PABC). Dipeptide Val-Cit is the most common cleavable linker in ADCs, exhibiting favorable plasma stability, release events, and chemical tractability [22]. MMAE does not accumulate easily in the eye. Therefore, the mechanism for anti-Nectin4-VcMMAE entry into the ocular cornea to induce toxicity is the key question of interest in the present study.

We aimed to investigate the molecular mechanisms of anti-Nectin4-VcMMAE-induced ocular toxicity. An in vivo rat model mimicking human ocular reactions and human corneal epithelial cell-transformed (HCE-T) cells was employed to clarify the key pathways for anti-Nectin4-VcMMAE entry into corneal cells and other factors affecting ocular toxicity. Additionally, potential molecular modifications to reduce the ocular toxicity of anti-Nectin4-VcMMAE were assessed. The results of this study will provide valuable insights into the reasonable design of ADCs to improve clinical safety.

## 2. Results

### 2.1. Establishment of the Anti-Nectin4-VcMMAE-Induced Ocular Toxicity Rat Model

**Figure 1:**
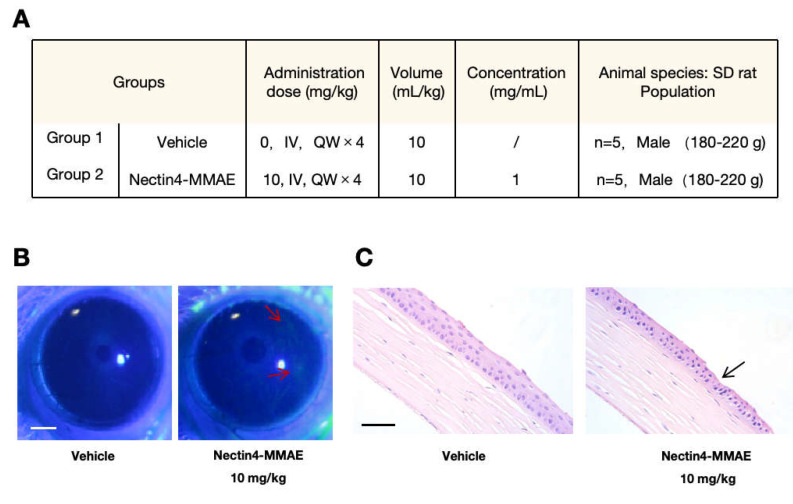
Establishing the anti-Nectin4-VcMMAE-induced ocular toxicity rat model. (**A**) Animal groups and drug administration. Rats were divided into two groups of 5. The drug was administered once a week for 4 weeks. (**B**) Representative figures showing sodium fluorescein staining of rat eyes. The right panel shows increased staining in the anti-Nectin4-VcMMAE group compared with staining in the control group. The area indicated by the red arrow in the figure represents the stained area, where green fluorescent signals can be observed. Scale bar = 1 mm. (**C**) Representative figure of ocular histopathological sections. The right panel shows increased corneal epithelial damage and cell necrosis in the anti-Nectin4-VcMMAE group compared with the control group. The area indicated by the black arrow in the figure is the part of the cornea with obvious damage. Scale bar = 200 μm.

Rats were chosen as the experimental animals because there were relevant preclinical studies on this ADC drug, and following a four-week injection, the rats exhibited ocular toxicity in the corneal region [18]. In preliminary experiments, intravenous injection of 0.5, 2, and 5 mg/kg anti-Nectin4-VcMMAE in male SD rats for 13 weeks induced mitotic abnormalities in the cornea and Harderian gland on histopathological examination. To verify that anti-Nectin4-VcMMAE could induce ocular toxicity, rats were treated with 10 mg/kg of the drug once per week for 4 weeks, and ocular status was evaluated to determine the induction of ocular toxicity. The control group was treated with intravenous injections of 5% glucose solution at an equal volume (Figure 1A).

To determine ocular status, sodium fluorescein staining was performed once a week. After 3 weeks, two rats in the experimental group exhibited significant staining compared with the control group (Figure 1B). The area indicated by the red arrow in Figure 1B represents the stained region, where green fluorescent signals can be observed. All rats were sacrificed after 4 weeks. The ocular pathological sections of the cornea of the two rats in the experimental group showed corneal degeneration and necrosis compared with the cornea in the control group (Figure 1C). The area indicated by the black arrow in Figure 1C is the part of the cornea with obvious damage. It can be seen that the edge of the tissue is serrated. Moreover, the cell nuclei in this area are significantly shrunken and show a deeper staining compared to those in the control group. These results verified the pathological model of anti-Nectin4-VcMMAE-induced ocular toxicity in rats.

### 2.2. Cytotoxicity and Receptor Expression in HCE-T Cells

**Figure 2:**
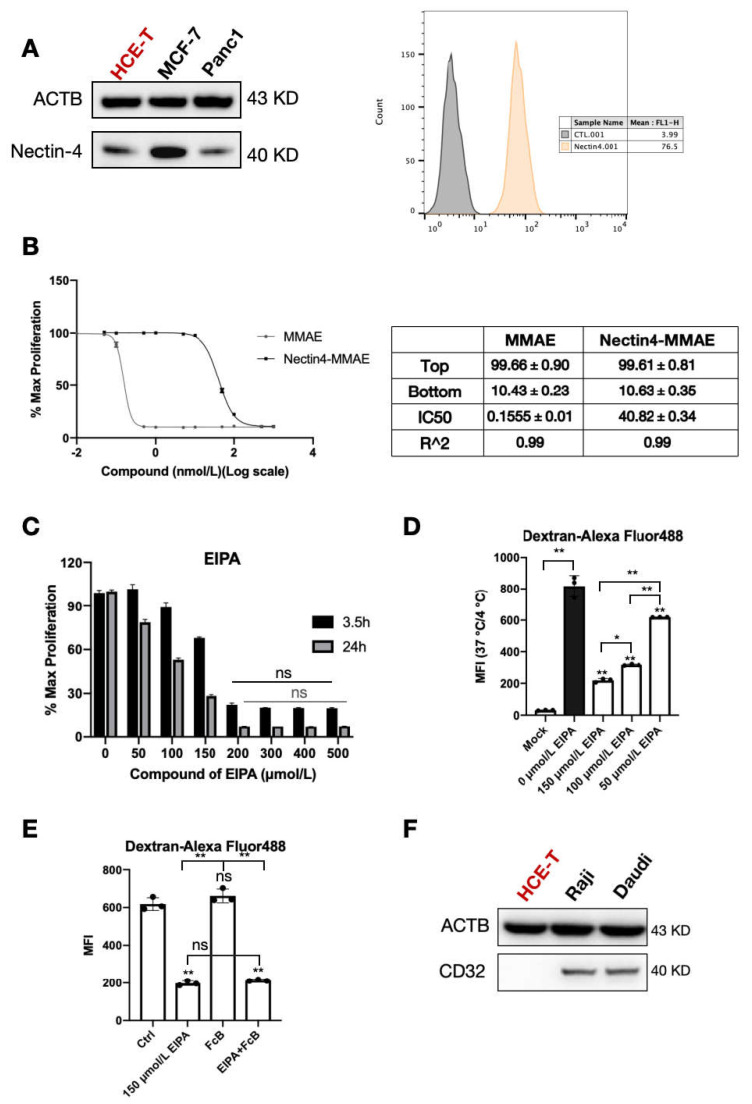
Cytotoxicity and receptor expression in HCE-T cells. (**A**) Western blot detection of Nectin-4 expression in the three cell types. ACTB (beta-actin) was used as a control (left). Flow cytometry detection of Nectin-4 expression in HCE-T cells. Control groups were unstained (right). (**B**) HCE-T cells were seeded into 96-well plates and incubated with gradient dilutions of MMAE and anti-Nectin4-VcMMAE for 4 days. After the addition of CCK-8, absorbance was measured at 450 nm (left). Several parameters were calculated, including upper plateau, lower plateau, IC50, and determination coefficient. Data are presented as the mean ± SEM (*n* = 3). (**C**) HCE-T cells were seeded into 96-well plates and treated with gradient dilutions of EIPA for 3.5 h (black bars) and 24 h (gray bars). After the addition of CCK-8, absorbance was measured at 450 nm. Data are presented as the mean ± SEM (*n* = 3). Except for the last four groups of data, there were significant differences between any two groups of data obtained at the same time point, with ns = non-significant (*p* > 0.05). (**D**) HCE-T cells were seeded into 6-well plates and treated with different concentrations of EIPA for 30 min at 37 °C, followed by the addition of 1 mg/mL Dextran-Alexa Fluor™ 488 for another 3 h. Cells were incubated at 4 °C for the Mock group. Intracellular fluorescence intensity was detected by flow cytometry. Data are presented as the mean ± SEM (*n* = 3), * *p* < 0.05, ** *p* < 0.01. (**E**) HCE-T cells were seeded into 6-well plates and treated with EIPA, FcB, or a combination of EIPA and FcB for 30 min, followed by the addition of 1 mg/mL Dextran-Alexa Fluor™ 488 for another 3 h. Intracellular fluorescence intensity was detected by flow cytometry. Data are presented as the mean ± SEM (*n* = 3), ** *p* < 0.01, ns = non-significant (*p* > 0.05). (**F**) Western blot detection of CD32 expression in the three cell types. ACTB was used as a control.

In vitro experiments using primary HCE-T cells were performed to detect the toxicity of anti-Nectin4-VcMMAE in corneal epithelium. Nectin-4 is the intracellular receptor for anti-Nectin4-VcMMAE. MCF-7 and Panc1 cells that express Nectin-4 were used as positive controls. Western blotting and flow cytometry showed that HCE-T cells express Nectin-4 (Figure 2A); thus, anti-Nectin4-VcMMAE may kill corneal epithelial cells via on-target-off tumor mechanisms. Cells were co-incubated with MMAE and ADC for 4 days. Based on the IC50s, the small molecule killed more cells than the whole ADC (Figure 2B). These findings also verified the adequate drug concentrations for subsequent experiments.

EIPA, a Na^+^/H^+^ exchange inhibitor, inhibits non-specific endocytosis [23,24,25]; thus, EIPA is cytotoxic. The cytotoxic effects of EIPA in HCE-T cells were detected. Three hours after treatment with 150 μM EIPA, 60% of cells were viable, and 24 h after treatment, cell viability was 30% (Figure 2C). To verify the inhibition of non-specific endocytosis, the effects of EIPA on the endocytosis of dextran were measured by flow cytometry; the mock group was a 4 °C control. Peak inhibitory activity was detected at 150 μM EIPA (Figure 2D). Therefore, 150 μM EIPA was selected for subsequent experiments.

The Fcγ receptor (FcγR) interacts with the Fc fragment of IgG antibodies. The FcγR is crucial for immune system effector activity and affects ADC entrance into cells [26,27]. The dependence of anti-Nectin4-VcMMAE endocytosis on the FcγR was investigated. An FcγR binding inhibitor polyclonal antibody (FcB) was used to block all three types of Fcγ receptors, including FcγRI (CD64), FcγRII (CD32), and FcγRIII (CD16). The endocytosis efficiency of dextran was affected by EIPA, but not FcB (Figure 2E). This result was consistent with the expected activity of EIPA [28]. FcγRI (CD64) is mainly expressed in monocytes and neutrophils, and FcγRIII (CD16) is predominantly expressed in macrophages and macrophage lineage cells, NK cells, bone marrow precursor cells, and neutrophil lineage cells. FcγRII (CD32) was not detected in HCE-T cells by Western blotting, indicating that HCE-T cells do not express FcγRII or the expression level was below the detection limitation (Figure 2F).

### 2.3. Cellular Entry Pathways for Anti-Nectin4-VcMMAE

**Figure 3:**
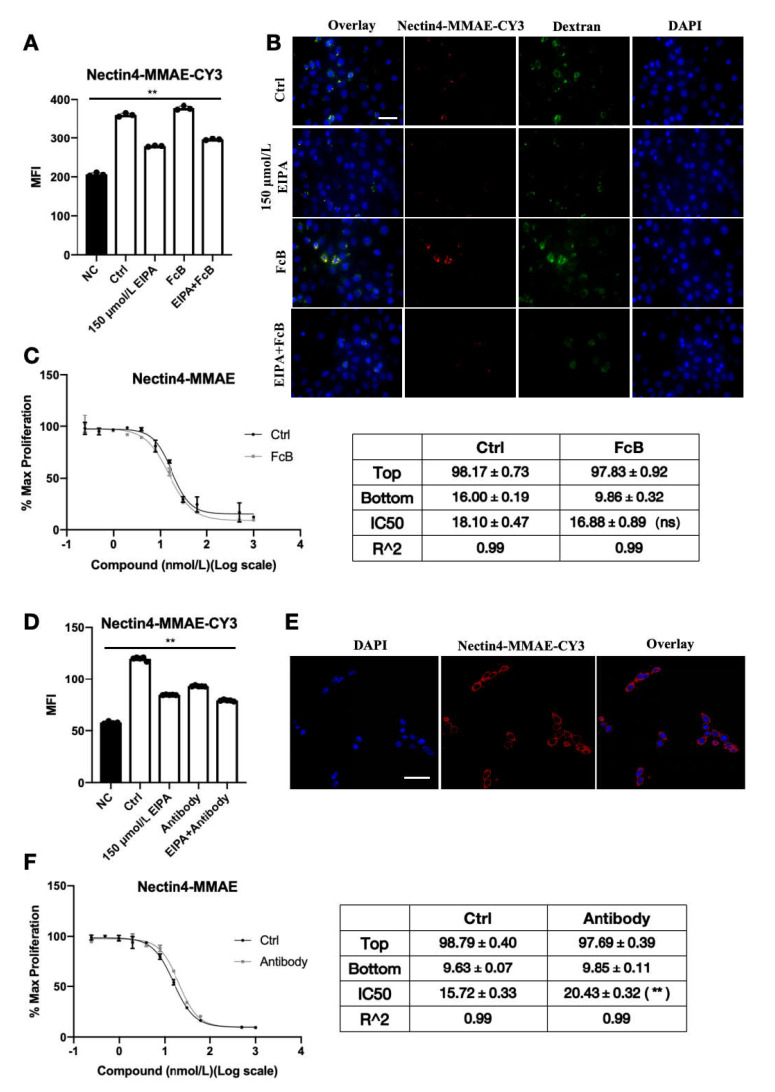
Cellular entry pathways for anti-Nectin4-VcMMAE. (**A**) HCE-T cells were seeded into 6-well plates and treated with EIPA, FcB, or a combination of EIPA and FcB for 30 min, followed by the addition of 40 μM anti-Nectin4-VcMMAE-CY3 for 3 h. Intracellular fluorescence was detected by flow cytometry. Data are presented as the mean ± SEM (*n* = 3). There were significant differences between any two groups of data, with ** *p* < 0.01. (**B**) HCE-T cells were seeded into the glass-bottom dishes and observed by fluorescence microscopy. Blue represents DAPI, red represents ADC inside cells, and green represents dextran. Scale bar = 50 μm. (**C**) HCE-T cells were seeded into 96-well plates. Cells in the treatment group were incubated with FcB for 30 min, followed by treatment with the indicated dilutions of anti-Nectin4-VcMMAE for 4 days. After the addition of CCK-8, absorbance was measured at 450 nm to detect surviving cells (left panel). Data are presented as the mean ± SEM (*n* = 3), ns = non-significant (*p* > 0.05). (**D**) HCE-T cells were treated with EIPA and 40 nM naked antibody for 30 min. After washing away the unbound antibody with PBS, anti-Nectin4-VcMMAE-CY3 was added to the cells. Flow cytometry was used to detect the endocytosis of ADC. Data are presented as the mean ± SEM (*n* = 4). There were significant differences between any two groups of data, with ** *p* < 0.01. (**E**) HCE-T cells were seeded into glass-bottom dishes and observed by fluorescence microscopy. Blue represents DAPI and red represents ADC in cells. Scale bar = 50 μm. (**F**) HCE-T cells were seeded into 96-well plates. Cells in the treatment group were incubated with naked antibody for 30 min, followed by the addition of gradient dilutions of anti-Nectin4-VcMMAE for 4 days. After the addition of CCK-8, absorbance was measured at 450 nm to detect surviving cells (left panel). Data are presented as the mean ± SEM (*n* = 3), ** *p* < 0.01.

The following experiments were conducted to determine how anti-Nectin4-VcMMAE enters corneal epithelial cells. The anti-Nectin4-VcMMAE-CY3 molecule was stably produced without affecting the physiological effects of ADC. Flow cytometry results showed that EIPA but not FcB inhibited the endocytosis of anti-Nectin4-VcMMAE (Figure 3A). Original flow cytometry data can be found in Supplementary S1. A similar trend was observed by fluorescence microscopy; EIPA significantly reduced the red and green fluorescence, indicating that ADC and dextran endocytosis were inhibited (Figure 3B). The expression level of Nectin4 in HCE-T cells is relatively low, and the toxicity of ADC is significantly manifested only at a relatively high concentration. We selected 50 nM as the ADC treatment concentration. At this concentration, we observed under the microscope that the internalization of Nectin4-MMAE-CY3 occurred in a few cells. The internalization status of different cells varies, and the fluorescence signals also differ greatly. The control group refers to the group without EIPA and FcB treatment, but only treated with Dextran-Alexa Fluor™488 and Nectin4-MMAE-CY3. It can be observed that the internalization of both Dextran-Alexa Fluor™ 488 and Nectin4-MMAE-CY3 is relatively obvious. After treating cells with EIPA, the red and green fluorescence signals decreased significantly, and the group treated with EIPA and FcB also showed a similar pattern. When the cells were treated with FcB alone, there was no obvious change in the signal intensity observed under the microscope. FcB did not affect the cell viability, consistent with previous results (Figure 3C).

The impact of Nectin-4 antigen expression was investigated next. Cells were treated with 40 nM naked antibody for 30 min, washed with PBS to remove unbounded antibody, and treated with anti-Nectin4-VcMMAE-CY3. The flow cytometry results demonstrated that naked antibody treatment reduced ADC endocytosis (Figure 3D). Original flow cytometry data can be found in Supplementary S2. After 30 min, most of the anti-Nectin4-VcMMAE-CY3 was bound at the surface of the cell membrane (Figure 3E). Treatment with naked antibody significantly increased the IC50 of anti-Nectin4-VcMMAE-induced HCE-T cell cytotoxicity (Figure 3F). These results indicate that the intracellular uptake of anti-Nectin4-VcMMAE was at least partially mediated by membrane Nectin-4.

### 2.4. Molecular Modification to Reduce Ocular Toxicity

**Figure 4:**
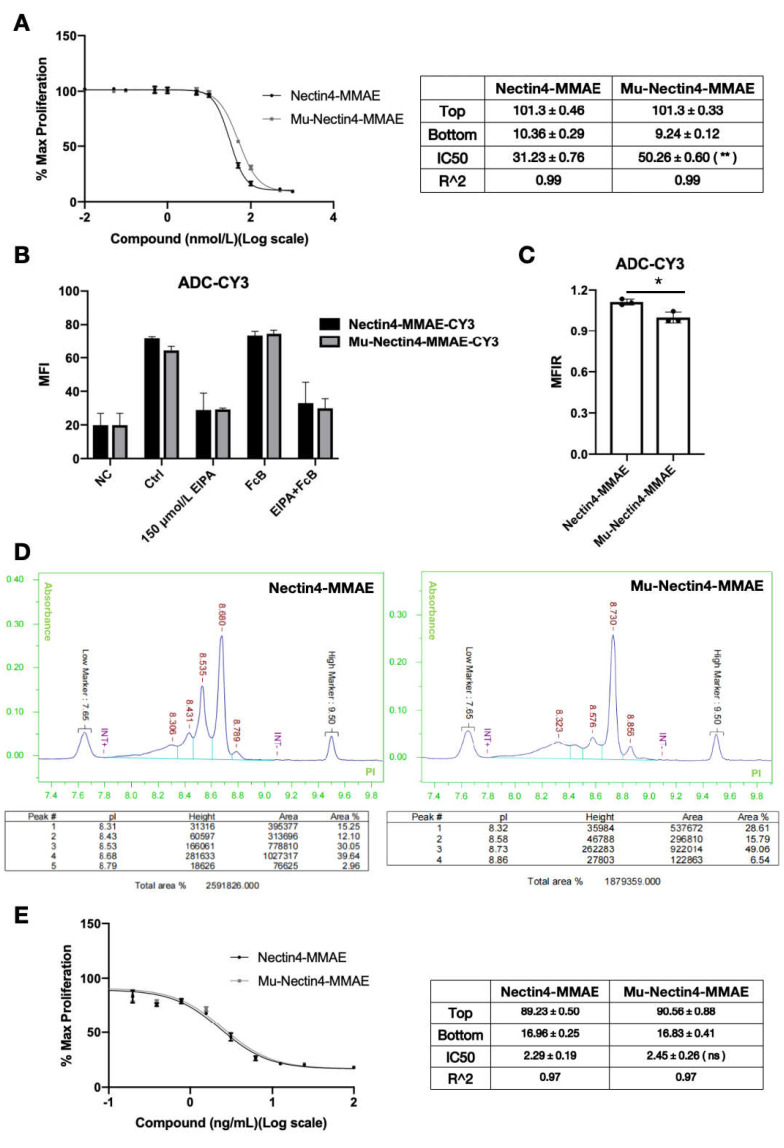
Molecular modification to reduce ocular toxicity. (**A**) HCE-T cells were seeded into 96-well plates and treated with gradient dilutions of anti-Nectin4-VcMMAE or Mu-anti-Nectin4-VcMMAE for 4 days. After the addition of CCK-8, absorbance was measured at 450 nm to detect surviving cells (left panel). Data are presented as the mean ± SEM (*n* = 3), ** *p* < 0.01. (**B**) HCE-T cells were treated with EIPA and FcB for 30 min, followed by the addition of either anti-Nectin4-VcMMAE-CY3 or mu-anti-Nectin4-VcMMAE-CY3. Flow cytometry was used to detect the endocytosis of ADC. Data are presented as the mean ± SEM (*n* = 3). (**C**) A statistical analysis of the results in Figure 4B comparing the endocytosis of anti-Nectin4-VcMMAE-CY3 vs. mu-anti-Nectin4-VcMMAE-CY3. Data are presented as the mean ± SEM (*n* = 3), * *p* < 0.05. (**D**) The charge heterogeneity of anti-Nectin4-VcMMAE (left) and mu-anti-Nectin4-VcMMAE (right) detected by iCIEF. The table shows the proportion of the area corresponding to each peak. The *x*-axis labeled “pI” stands for “isoelectric point”. It is the pH value at which a molecule has a net charge of zero. Each peak corresponds to a specific charge-state species with its own pI value, and the table shows the proportion of the area of each peak, which reflects the relative abundance of each charge-state species. (**E**) PC3-AGS22 cells were seeded into 96-well plates and treated with gradient dilutions of anti-Nectin4-VcMMAE or mu-anti-Nectin4-VcMMAE for 4 days. After the addition of CCK-8, absorbance was measured at 450 nm to detect surviving cells (left panel). Data are presented as the mean ± SEM (*n* = 3), ns = non-significant (*p* > 0.05).

A mutated molecule was designed to reduce the ocular toxicity of anti-Nectin4-VcMMAE. The Fc fragment of anti-Nectin4-VcMMAE was mutated to remove the antibody-dependent cell-mediated cytotoxicity and complement-dependent cytotoxicity. Cell viability and the IC50 were significantly higher after treatment with Mu-anti-Nectin4-VcMMAE compared with the control groups, indicating that the mutation decreased cytotoxicity (Figure 4A). The endocytosis characteristics of Mu-anti-Nectin4-VcMMAE and anti-Nectin4-VcMMAE were similar; endocytosis of both molecules was affected by EIPA but not FcB treatment (Figure 4B). However, endocytosis of Mu-anti-Nectin4-VcMMAE was reduced compared with the endocytosis of anti-Nectin4-VcMMAE (Figure 4C). Although the mutated ADC demonstrated significantly reduced toxicity in in vitro experiments, its in vivo effects still require further validation. Currently, the amount of purified drug is insufficient for animal studies, and we plan to conduct corresponding research in subsequent phases.

Alterations in charge heterogeneity may affect non-specific pinocytosis [25]. We hypothesized that the decline of endocytosis was due to altered charge heterogeneity. The iCIEF results showed that the charge heterogeneity of Mu-anti-Nectin4-VcMMAE was significantly different from the charge heterogeneity of anti-Nectin4-VcMMAE (Figure 4D). The differences in the number, position, and relative intensity of the peaks between Nectin4-MMAE and Mu-Nectin4-MMAE indicate significant differences in their charge heterogeneity. Charge heterogeneity can affect the physical and chemical properties of antibody-drug conjugates, such as solubility and binding affinity. The distinct peak patterns suggest that the charge-state species of Mu-Nectin4-MMAE are different from those of Nectin4-MMAE, which may further influence their biological functions, such as endocytosis, consistent with our hypothesis about the relationship between charge heterogeneity and endocytosis decline. In PC3-AGS22 cells stably expressing Nectin-4, no significant differences in cell viability were detected between Mu-anti-Nectin4-VcMMAE and anti-Nectin4-VcMMAE (Figure 4E). These results demonstrate that ADC molecules with point mutations in the Fc fragment exhibit lower toxicity in vitro, and the decreased toxicity may be related to altered charge heterogeneity that affects non-specific endocytosis. Certainly, there are numerous factors influencing endocytosis efficiency, and charge properties represent only one factor. We will further explore more potential causes in our subsequent studies.

## 3. Discussion 

The mechanisms of enfortumab vedotin-induced ocular toxicity were examined. Our results clarify the mechanisms of ADC-induced ocular toxicity and lay a strong foundation for subsequent drug design optimization with translational potential. The cornerstone of the present study is the successful establishment of a rat model of enfortumab vedotin-induced ocular toxicity. The ocular structure and sensitivity in rats are more like humans than rabbits and *Macaca fascicularis*; thus, the possible side effects of enfortumab vedotin in rats are similar to the side effects in humans. Degeneration and necrosis of the corneal epithelium were observed in rats after weekly intravenous injection of 10 mg/kg enfortumab vedotin for 4 weeks. The ocular surface damage, as shown by sodium fluorescein staining, was consistent with the cornea-related pathology described in clinical reports in patients treated with enfortumab vedotin. Our rat model of enfortumab vedotin-induced ocular toxicity provides a valid platform for subsequent in-depth studies on the entry of drugs and drug-induced toxicity, filling the gap between the clinical phenotype and fundamental mechanistic research.

ADC drugs exert cytotoxicity via two mechanisms, on-target-off-tumor and off-target-off-tumor. The on-target-off-tumor mechanism occurs when the antibody-targeted antigen is expressed in other sites besides the tumor; ADC targets normal organs, leading to cytotoxic effects. The second mechanism, which is more common, is the off-target-off-tumor mechanism [29]. On-target-off-tumor effects are mediated by receptors on the cell surface, including FcγR, neonatal Fc receptors, and C-type lectin receptors. Non-specific endocytosis, such as macropinocytosis and micropinocytosis, also contributes to toxicity. The endocytosis efficiency correlates with the charge heterogeneity, i.e., the hydrophilicity/hydrophobicity [23,24,25,26]. Additionally, if an unstable linker is used in the ADC, the small molecule may break from the antibody before entering cells via passive diffusion [30]. The bystander effect may also contribute to cytotoxicity. After ADC kills cells, cell membrane disruption releases the lysed antibody and small molecules, which enter cells via passive diffusion [31]. Our results demonstrate that enfortumab vedotin enters corneal epithelial cells via the Nectin-4-mediated on-target-off tumor mechanism and non-specific endocytosis.

Nectin-4 is stably expressed in the corneal epithelial cell HCE-T lineage. When nectin-4 was pre-blocked with naked antibody, the endocytosis efficiency of ADC declined significantly, and the cytotoxic IC50 increased significantly. Fluorescence microscopy showed that ADC binds to the cell membrane surface. These results provide conclusive evidence that Nectin-4 plays a central role in enfortumab vedotin uptake by HCE-T cells. As an antigen, enfortumab vedotin binds specifically to cell surface Nectin-4 to form a complex and initiate endocytosis, allowing the drug to enter cells and exert its effects. This process is similar to the initial step of targeted killing of tumor cells but causes ocular toxicity.

Non-specific pinocytosis may facilitate enfortumab vedotin entry into corneal epithelial cells. In the present study, enfortumab vedotin and dextran endocytosis were significantly inhibited by EIPA, indicating that non-specific pinocytosis contributes to the entry of enfortumab vedotin into HCE-T cells. Although the relative contribution of pinocytosis is difficult to quantify, the presence of pinocytosis indicates that multiple redundant mechanisms are involved in the cellular uptake of drugs. Thus, drugs may still enter corneal epithelial cells if one pathway is blocked, highlighting the complexity of ocular toxicity.

To reduce the ocular toxicity of enfortumab vedotin, we constructed ADC variants with point mutations in the Fc fragment, referred to as Mu-anti-Nectin4-VcMMAE. The cytotoxicity of Mu-anti-Nectin4-VcMMAE in corneal cells was lower than the cytotoxicity of anti-Nectin4-VcMMAE without mutations. In PC3-AGS22 cells stably expressing Nectin-4, the biological activity of the mutated ADC was similar to the un-mutated drug, indicating that Mu-anti-Nectin4-VcMMAE retained the targeted anti-tumor activity but ocular toxicity was reduced. Endocytosis of the mutant molecule was consistent with the unmutated drug but significantly reduced. In addition, iCIEF showed that the charge heterogeneity of the mutated drug was altered. Therefore, altered charge distribution due to point mutations of the Fc fragment may have affected non-specific interactions with the cell membrane, resulting in reduced non-specific pinocytosis and, consequently, reduced unnecessary ocular uptake of the drug. Our results highlight a new strategy for ADC drug design to low ocular toxicity.

The mechanisms of enfortumab vedotin-induced ocular toxicity revealed in the present study provide theoretical guidance for the rational design of ADC drugs. Given the involvement of non-specific pinocytosis, future research should focus on modifying the physicochemical properties of ADC molecules, including adjusting charge and hydrophilicity/hydrophobicity to reduce non-specific cellular uptake. Point mutation of the Fc fragment provides an example of engineering ADC modifications to balance immunomodulation and toxicity.

For patients currently using or about to use ADC drugs such as enfortumab vedotin, physicians should be aware of the occurrence of ocular toxicity, particularly in patients undergoing long-term or high-dosage treatments. Routine ocular detection should include regular monitoring to identify early ocular pathogenesis and facilitate timely intervention. Once ocular toxicity is diagnosed, adjusting the dosage and frequency should be considered to minimize the effects of ocular toxicity on the quality of life.

Although the present study helps clarify the mechanism of ADC-mediated ocular toxicity, several study limitations should be considered. First, animal models may mimic human status but there are taxonomic differences. The dynamic processes, long-term impact, and individual variations in enfortumab vedotin-induced ocular toxicity in humans are impossible to fully recapitulate in a rat model. In future studies, human ocular tissue specimens or organoids should be used to mimic the real condition. Second, investigations into the mechanisms of Fc fragment point mutations in reducing ocular toxicity were limited to the charge heterogeneity. In-depth molecular structure-function studies using techniques such as cryo-electron microscopy and molecular dynamic simulations should be conducted to guide mutation design. Overall, our results clarify the mechanism of ADC-induced ocular toxicity. Future investigations should consider integrating multi-disciplinary techniques to balance the efficacy and safety of ADC drugs.

## 4. Materials and Methods

### 4.1. Cell Culture

HCE-T cells (Cat# CL-0743) and culture medium (Cat# CM-0743) were purchased from Wuhan Pricella Biotechnology Co., Ltd. (Wuhan, China). MCF-7, Panc1, Raji, and Daudi cells were purchased from the American Type Culture Collection (Manassas, VA, USA). MCF-7 and Panc1 cells were cultured in DMEM (Cat#SH30243.01, Hyclone, Logan, UT, USA) containing 10% fetal bovine serum (Cat#16000077, Gibco, Waltham, MA, USA). Raji and Daudi cells were cultured in RPMI 1640 Medium (Life Technologies, Waltham, MA, USA, Cat# 21875-034) with 10% fetal bovine serum (Gibco). PC3-AGS22 cells, which are human prostate cancer cells stably expressing Nectin-4, were purchased from Astellas Pharma Inc. (Tokyo, Japan). and cultured in RPMI 1640 Medium (Life Technologies, Cat# 21875-034) with 10% FBS (Gibco). All cells were cultured at 37 °C with 5% CO_2_. When the cell density exceeds 80%, trypsin (Gibco) will be used for subculture treatment.

### 4.2. Construction of the ADC

Anti-Nectin4-VcMMAE (product number 166211, batch number 4001867, 30 mg specification) was provided by Astellas. During the experiment, it was still within the expiration date. It was stored in a dry powder state at 4 °C and diluted with normal saline before use. According to the instruction manual, its drug–antibody ratio (DAR) is between 3.7 and 4.1. The anti-Nectin4 antibody was provided by Agensys (batch number DT1583, 1.77 mg/mL, Shohola, PA, USA). It was aliquoted and stored at −80 °C. After thawing, it should be immediately stored at 2–8 °C and can be stored for up to 9 weeks. MMAE was purchased from MedChemExpress (Cat# HY-15162, Monmouth Junction, NJ, USA).

Anti-Nectin4-VcMMAE with point mutations in the Fc fragment was produced by stably expressing the point mutation-containing heavy and light chain in hamster ovary cells and harvesting the antibody.

Experimental Details of the Conjugation Process: The antibody buffer system was replaced with PBS7.0/EDTA, and the antibody solution was prepared at a concentration of 10 mg/mL. This solution was placed in an EP tube, and 10 mM TCEP aqueous solution (2.5 equivalents relative to one molecule of antibody) was added to it. The mixture was incubated at 30 °C for 1 h to reduce the interchain disulfide bonds of the antibody.

At room temperature, a 10 mM DMSO solution of vcMMAE (5 equivalents relative to one molecule of antibody) was added to the above solution, and the mixture was thoroughly mixed. The reaction was allowed to proceed at room temperature for 30 min to conjugate vcMMAE to the antibody. Subsequently, a 100 mM L-cysteine hydrochloride solution (3 equivalents relative to one molecule of antibody) was added, and then the reaction was allowed to continue at room temperature for 20 min to terminate the conjugation reaction. The mutated ADC (referred to as Mu-anti-Nectin4-VcMMAE) was chemically conjugated to the small molecule MMAE via a Val-Cit linker to produce the final ADC drug with a drug-to-antibody ratio (DAR) of 3.8.

Anti-Nectin4-VcMMAE-CY3 and Mu-anti-Nectin4-VcMMAE-CY3 were prepared and provided by the laboratory of Dr. Peng Guo from the Chinese Academy of Sciences, Hangzhou. Anti-Nectin4-VcMMAE was covalently coupled to the CY3 molecule to form a stable complex. To prepare the fluorescently labeled ADCs, 1 mg of ADC was reacted with Cyanine3 N-hydroxysuccinimide ester (CY3-NHS ester) dye at a molar ratio of 1:5 (antibody: CY3). The reaction was performed in PBS buffer (pH 8.5) to a final volume of 500 μL and incubated at room temperature for 4 h. After the reaction, unbound CY3 dye was removed by purification through a PD MiniTrap G-25 column (Cat#28918007, Cytiva, Washington, DC, USA). Both Anti-Nectin4-Vc-MMAE-CY3 and Mu-Anti-Nectin4-Vc-MMAE-CY3 conjugates were successfully prepared.

The analytical data related to the synthesized ADCs, including their purity and drug-antibody ratio (DAR), can be found in the Supplementary S3–S6.

### 4.3. Western Blotting

After reaching 90% confluence, cells were lysed in ice-cold RIPA buffer (Cat# 89901, Thermo Scientific, Waltham, MA, USA) containing protease inhibitors (Thermo Scientific, Cat# 78430). The cell lysate protein concentration was measured using a BCA Protein Assay Kit (Thermo Scientific, Cat# 23225). Proteins from cell lysates were separated on 10% SDS-PAGE gels and transferred to PVDF membranes. Subsequently, the membrane was blocked with 5% skim milk for 1 h. After incubating with primary and secondary antibodies, antibody binding on the membranes was detected using the ImageQuant LAS image system (GE, Marlborough, MA, USA). Primary antibodies included beta-actin (13E5) rabbit mAb (Cat# 4970S, CST, Danvers, MA, USA), recombinant anti-nectin-4 antibody (Cat# ab192033, Abcam, Cambridge, UK), and anti-CD32 antibody (Abcam, Cat# ab155972). The secondary antibody was goat anti-rabbit IgG (H + L) conjugated to HRP (Thermo Scientific, Cat# 31460). The dilution ratios of both the primary antibody and the secondary antibody were operated according to the instructions in the manual. The chemiluminescent luminescent solution and enhancement solution were SuperSignal™ West Pico PLUS (Thermo Scientific, Cat# 34577).

### 4.4. Flow Cytometry to Detect Nectin-4 Expression

Cells were seeded into 6-well plates and collected after 24 h of culture. The cells were treated with 50 nM anti-Nectin4-VcMMAE on ice for one hour. Cells were washed twice with PBS and stained with goat anti-human IgG (H + L) cross-adsorbed secondary antibody, Alexa Fluor™ 488 (Thermo Scientific, A-11013) for 30 min with the dilution ratio of 1:1000. The fluorescent signal was detected through the FL1 channel by using a BD FACS-Calibur flow cytometer (BD Bioscience, Franklin Lakes, NJ, USA). Data were analyzed using Flowjo 10 software.

### 4.5. Dose-Response Relationship

HCE-T cells (100 μL) were seeded into 96-well plates (Cat#354650, Corning, New York, NY, USA) at 1000 cells per well. After 8–12 h, different concentrations of ADC and small molecules were added. ADC and small molecules have a total of 12 concentration points: 0, 0.01, 0.05, 0.1, 0.5, 1, 5, 10, 50, 100, 500, and 1000 nM. After 4 days of treatment, the culture medium was removed and 100 μL of medium-diluted CCK-8 (Cat# CA1210-500T, Solarbio, Beijing, China) was added. After culturing cells for 4 h at 37 °C, the absorbance at 450 nm was measured using a microplate reader (Molecular Devices, San Jose, CA, USA, Spectramax M5). Data were normalized to the control data and analyzed in GraphPad Prism 8. The IC50 value was obtained using the log (inhibitor) vs. response with a variable slope (four parameters).

PC3-AGS22 cells were detected in a similar manner. On the first day, the cells were seeded at a density of 1.5 × 10^4^ cells/mL, with 100 μL per well. On the second day, Nectin4-MMAE and Mu-Nectin4-MMAE were diluted and added to the assay plate. The diluted concentrations of the samples were 100, 25, 12.5, 6.25, 3.125, 1.563, 0.781, 0.391, 0.195, and 0.049 ng/mL, respectively. The assay plate was incubated for 120 h. On the seventh day, 20 μL of PrestoBlue^®^ reagent (Life Technologies, Cat# A13262) was added to each well, and the microplate was incubated for another 2 h ± 30 min. The reduction of the dye could be determined by fluorescence measurement at an excitation wavelength of 540 nm and an emission wavelength of 590 nm. The dose–response curve was obtained through a four-parameter logistic fitting model, which plotted the graph with the relative fluorescence units (RFU) as the ordinate and the logarithmic concentration of the test sample as the abscissa. The relative potency of the test sample was calculated as the ratio of the C parameters derived from the four-parameter logistic fitting model.

### 4.6. Fluorescence Microscopy and Flow Cytometry Detection of ADC Endocytosis Efficiency

HCE-T cells (3 × 10^5^ cells) were seeded into glass-bottom dishes (Cat# 150680, Nunc, Roskilde, Denmark). After culturing for 24 h, cells were treated with 150 μM ethylisopropylamiloride (EIPA) (Cat# HY-101840, MedChemExpress, NJ, USA) and Fc receptor binding inhibitor polyclonal antibody (Cat# 16-9161-73, eBioscience™, Santa Clara, CA, USA) with the dilution ratio of 1:5 for 30 min. Anti-Nectin4-VcMMAE-CY3 (50 nM) and 1 mg/mL Dextran-Alexa Fluor™ 488 (10,000 MW, Life Technologies, Cat# D22910) were added and the cells were cultured at 37 °C for 3 h. After washing with PBS, cells were fixed with 1 mL 4% paraformaldehyde (Cat# abs9179, absin, Shanghai, China) at room temperature for 20 min. Anti-fade mounting medium (ProLong™ Diamond, Thermo Scientific, Cat# P36962) containing 4′,6-diamidino-2-phenylindole (DAPI) was added to the cells. Cells were imaged using fluorescence microscopy (Nikon, Tokyo, Japan, Ts2R). The intracellular fluorescence intensity of ADC-CY3 was detected through the FL2 channel with a FACS-Calibur flow cytometer (BD Bioscience) and data were analyzed using Flowjo 10 software.

### 4.7. Imaged Capillary Isoelectric Focusing (iCIEF) Assay

Imaged capillary isoelectric focusing (iCIEF) is a separation technique. In the iCIEF process, a capillary is filled with a mixture of carrier ampholytes and the sample. When an electric field is applied, the ampholytes migrate to form a pH gradient within the capillary. The sample components then migrate to their respective isoelectric points in this gradient and focus. After focusing, the capillary is scanned, and the distribution of sample components is detected, generating a profile of charge heterogeneity.

The following chemicals were used: 1% methyl cellulose solution (Cat# 101876, Protein Simple, San Jose, CA, USA), 0.5% methyl cellulose solution (Protein Simple, Cat# 102505), Pharmalyte 8-10.5 (Cat# 17-0455-01, GE Healthcare Biosciences, Natick, MA, USA), Pharmalyte 5-8 (GE Healthcare, Cat# 17-0453-01), pI marker 7.65 (Protein Simple, Cat# 102407), pI marker 9.50 (Protein Simple, Cat# 101996), electrolyte kit (Protein Simple, Cat# 102506), 10 mM phosphate-buffered saline (pH 6.5), and 35 mM citric acid. To prepare the zwitterionic electrolyte solution, 1.20 g urea was dissolved in 1000 µL of 35 mM citric acid, 300 µL water, 2250 µL of 1% methyl cellulose solution, 100 µL Pharmalyte 5–8, 100 µL Pharmalyte 8-10.5, 25 µL pI marker 7.65, and 25 µL pI marker 9.50. Water was added to the volumetric flask to bring the volume to 5 mL. The test sample was diluted in 10 mM phosphate-buffered saline (pH 6.5). The samples were loaded, and data were collected and analyzed using a Protein Simple iCE3 analyzer.

### 4.8. Animal Experiment

Sprague-Dawley (SD) rats were intravenously injected with 10 mg/kg anti-Nectin4-VcMMAE. Each group consisted of 5 male SD rats, with a body weight of 180–220 g. The solvent control group received an equal volume of 5% glucose injection. The drug was administered once per week for 4 weeks. After treatment, 2 µL of 0.2% sodium fluorescein solution was instilled into the inferior conjunctival sac of the rat. The eyelid was gently closed for approximately 2 s and then rinsed with saline. The staining level of the ocular surface was examined under a slit-lamp microscope with cobalt blue diffuse light. After 4 weeks, eyes and optic nerves were collected, paraffin-embedded, hematoxylin–eosin stained, and examined under a microscope.

### 4.9. Data Analysis

All data were represented as the mean ± SEM. The experiments were performed at least three times. The one-way ANOVA test was used for analysis between groups. *p* < 0.05 indicates the distinction is significant.

## Data Availability

Data is contained within the article and Supplementary Materials.

## Supplementary Materials

The following supporting information can be downloaded at: https://www.mdpi.com/article/10.3390/ijms26114996/s1.

## References

[1] Moolten F.L., Capparell N.J., Cooperband S.R. (1972). Antitumor effects of antibody-diphtheria toxin conjugates: Use of hapten-coated tumor cells as an antigenic target. J. Natl. Cancer Inst..

[2] Beck A., Reichert J.M. (2013). Antibody-Drug Conjugates. Proc. Natl. Acad. Sci. USA.

[3] Jaffry M., Choudhry H., Aftab O.M., Dastjerdi M.H. (2023). Antibody-Drug Conjugates and Ocular Toxicity. J. Ocul. Pharmacol. Ther..

[4] Carter P.J., Senter P.D. (2013). Antibody-drug conjugates in cancer therapy. Annu. Rev. Med..

[5] Li C., Wang J., Wang Y., Gao H., Wei G., Huang Y., Yu H., Gan Y., Wang Y., Mei L. (2019). Recent progress in drug delivery. Acta Pharm. Sin. B.

[6] Zhao P., Zhang Y., Li W., Jeanty C., Xiang G., Dong Y. (2020). Recent advances of antibody drug conjugates for clinical applications. Acta Pharm. Sin. B.

[7] Rowland G.F., O’Neill G.J., Davies D.A.L. (1975). Suppression of tumour growth in mice by a drug-antibody conjugate using a novel approach to linkage. Nature.

[8] Moolten F.L., Cooperband S.R. (1970). Selective destruction of target cells by diphtheria toxin conjugated to antibody directed against antigens on the cells. Science.

[9] Eaton J.S., Miller P.E., Mannis M.J., Murphy C.J. (2015). Ocular Adverse Events Associated with Antibody-Drug Conjugates in Human Clinical Trials. J. Ocul. Pharmacol. Ther..

[10] Zhou L., Wei X. (2021). Ocular Immune-Related Adverse Events Associated with Immune Checkpoint Inhibitors in Lung Cancer. Front. Immunol..

[11] Wahab A., Rafae A., Mushtaq K., Masood A., Ehsan H., Khakwani M., Khan A. (2021). Ocular Toxicity of Belantamab Mafodotin, an Oncological Perspective of Management in Relapsed and Refractory Multiple Myeloma. Front. Oncol..

[12] Farooq A.V., Degli Esposti S., Popat R., Thulasi P., Lonial S., Nooka A.K., Jakubowiak A., Sborov D., Zaugg B.E., Badros A.Z. (2020). Correction to: Corneal Epithelial Findings in Patients with Multiple Myeloma Treated with Antibody-Drug Conjugate Belantamab Mafodotin in the Pivotal, Randomized, DREAMM-2 Study. Ophthalmol. Ther..

[13] Raheem F., Alsuhebany N., Zacholski E.H., Paulic N., Sandler A., Uk N., Moore D.C. (2023). Ocular toxicities associated with antibody drug conjugates and immunotherapy in oncology: Clinical presentation, pathogenesis, and management strategies. Expert Opin. Drug Saf..

[14] Astellas Pharma Inc., Seagen Inc. (2023). PADCEV (enfortumab vedotin-ejfv) [lable], US 2023.

[15] Rosenberg J.E., O’Donnell P.H., Balar A.V., McGregor B.A., Heath E.I., Yu E.Y., Galsky M.D., Hahn N.M., Gartner E.M., Pinelli J.M. (2019). Pivotal Trial of Enfortumab Vedotin in Urothelial Carcinoma After Platinum and Anti-Programmed Death 1/Programmed Death Ligand 1 Therapy. J. Clin. Oncol..

[16] Powles T., Valderrama B.P., Gupta S., Bedke J., Kikuchi E., Hoffman-Censits J., Iyer G., Vulsteke C., Park S.H., Shin S.J. (2024). Enfortumab Vedotin and Pembrolizumab in Untreated Advanced Urothelial Cancer. N. Engl. J. Med..

[17] Powles T., Rosenberg J.E., Sonpavde G.P., Loriot Y., Durán I., Lee J.-L., Matsubara N., Vulsteke C., Castellano D., Wu C. (2021). Enfortumab Vedotin in Previously Treated Advanced Urothelial Carcinoma. N. Engl. J. Med..

[18] U.S. Food and Drug Administration Drugs@FDA [Internet Database] BLA761137 Multi-Discipline Review. https://www.accessdata.fda.gov/drugsatfda_docs/nda/2019/761137Orig1s000TOC.cfm.

[19] Del Monte D.W., Kim T. (2011). Anatomy and physiology of the cornea. J. Cataract. Refract. Surg..

[20] Lindgren E.S., Yan R., Cil O., Verkman A.S., Chan M.F., Seitzman G.D., Farooq A.V., Huppert L.A., Rugo H.S., Pohlmann P.R. (2024). Incidence and Mitigation of Corneal Pseudomicrocysts Induced by Antibody-Drug Conjugates (ADCs). Curr. Ophthalmol. Rep..

[21] Loberg L.I., Henriques T.A., Johnson J.K., Miller P.E., Ralston S.L. (2022). Characterization and Potential Mitigation of Corneal Effects in Nonclinical Toxicology Studies in Animals Administered Depatuxizumab Mafodotin. J. Ocul. Pharmacol. Ther..

[22] Lyon R.P., Bovee T.D., O Doronina S., Burke P.J., Hunter J.H., Neff-LaFord H.D., Jonas M., E Anderson M., Setter J.R., Senter P.D. (2015). Reducing hydrophobicity of homogeneous antibody-drug conjugates improves pharmacokinetics and therapeutic index. Nat. Biotechnol..

[23] Zhao H., Gulesserian S., Ganesan S.K., Ou J., Morrison K., Zeng Z., Robles V., Snyder J., Do L., Aviña H. (2017). Inhibition of Megakaryocyte Differentiation by Antibody-Drug Conjugates (ADCs) is Mediated by Macropinocytosis: Implications for ADC-induced Thrombocytopenia. Mol. Cancer Ther..

[24] Ait-Oudhia S., Zhang W., Mager D.E. (2017). A Mechanism-Based PK/PD Model for Hematological Toxicities Induced by Antibody-Drug Conjugates. AAPS J..

[25] Zhao H., Atkinson J., Gulesserian S., Zeng Z., Nater J., Ou J., Yang P., Morrison K., Coleman J., Malik F. (2018). Modulation of Macropinocytosis-Mediated Internalization Decreases Ocular Toxicity of Antibody-Drug Conjugates. Cancer Res..

[26] Wang H.-M., Jeng J.-E., Kaplan H.J. (1989). Fc receptors in corneal epithelium. Curr. Eye Res..

[27] Carr D.J., Berube A.N., Filiberti A., Gmyrek G.B. (2021). Lack of neonatal Fc receptor does not diminish the efficacy of the HSV-1 0DeltaNLS vaccine against ocular HSV-1 challenge. Vaccine.

[28] Zheng Z., Pan X., Luo L., Zhang Q., Huang X., Liu Y., Wang K., Zhang Y. (2022). Advances in oral absorption of polysaccharides: Mechanism, affecting factors, and improvement strategies. Carbohydr. Polym..

[29] Tarcsa E., Guffroy M.R., Falahatpisheh H., Phipps C., Kalvass J.C. (2020). Antibody-drug conjugates as targeted therapies: Are we there yet? A critical review of the current clinical landscape. Drug Discov. Today Technol..

[30] Drago J.Z., Modi S., Chandarlapaty S. (2021). Unlocking the potential of antibody-drug conjugates for cancer therapy. Nat. Rev. Clin. Oncol..

[31] Erickson H.K., Widdison W.C., Mayo M.F., Whiteman K., Audette C., Wilhelm S.D., Singh R. (2010). Tumor delivery and in vivo processing of disulfide-linked and thioether-linked antibody-maytansinoid conjugates. Bioconjug. Chem..

