# Supplementary material for: Mechanistic Insights into Anti-Nectin4-VcMMAE-Induced Ocular Toxicity: From Cellular Uptake Pathways to Molecular Modification
Source: Int J Mol Sci. 2025 May 22;26(11):4996. doi: 10.3390/ijms26114996 (PMC12154390; doi:10.3390/ijms26114996)
Supplement: Supplementary file 1 [file ijms-26-04996-s001.zip › ijms-3574138-supplementary.pdf]

S1

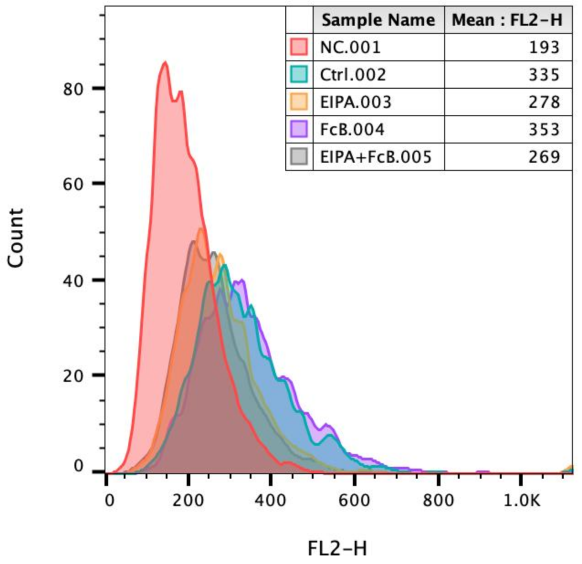

S2

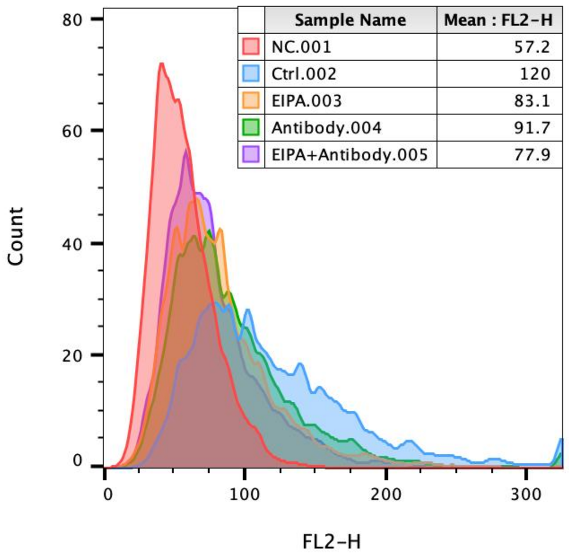

(S1) HCE-T cells were seeded into 6-well plates and treated with EIPA, FcB, or a combination of EIPA and FcB for 30 min, followed by the addition of 40 μM anti-Nectin4-VcMMAE-CY3 for 3 hours. Intracellular fluorescence was detected by flow cytometry.

(S2) HCE-T cells were treated with EIPA and 40 nM naked antibody for 30 min. After washing away the unbound antibody with PBS, anti-Nectin4-VcMMAE-CY3 was added to the cells. Flow cytometry was used to detect the endocytosis of ADC. Data are presented as the mean ± SEM (n = 4).

S3

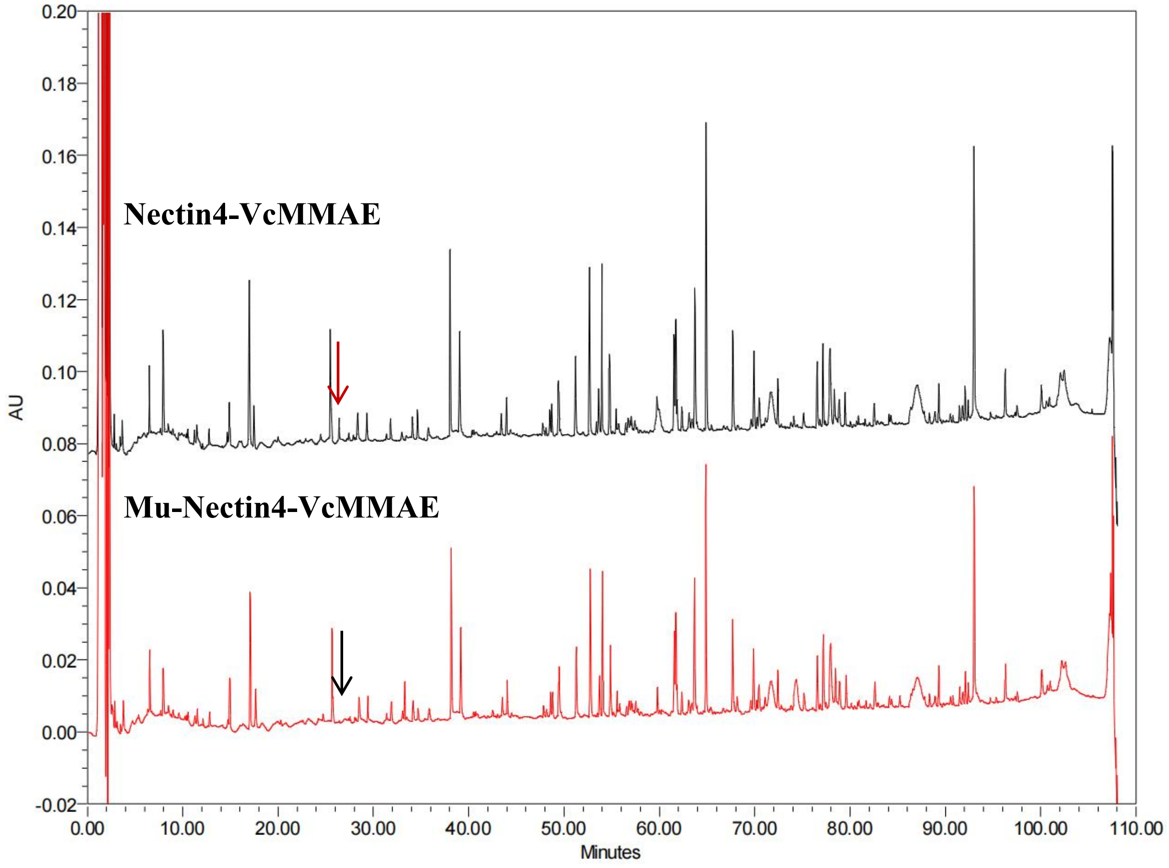

(S3) The peptide maps of Nectin4-VcMMAE and mu-Nectin4-VcMMAE are shown in the figure. The positions indicated by the arrows are the mutation sites of the antibody.

Peptide Mapping Method: Take 100 μg of the sample, add 8M guanidine hydrochloride solution to a final concentration of 6M, add 1M dithiothreitol (DTT) to a final concentration of 20 mM, mix well, react at 56 ˚C for 30 minutes. After cooling to room temperature, add 1M iodoacetamide (final concentration 50 mM), mix well, and react in the dark at room temperature for 45 minutes. Add 400 μL of 2M urea (Tris-HCl, pH 7.5) to the upper layer of a 10 kDa ultrafiltration membrane, centrifuge at 13,000 rpm for 10 minutes, discard the waste liquid. Add the alkylated sample to the 10 kDa ultrafiltration membrane and supplement with 2M urea (Tris-HCl, pH 7.5) to a volume of 400 μL, mix well, centrifuge at 13,000 rpm for 10 minutes, discard the waste liquid, and repeat the replacement twice. Add Trypsin enzyme according to the ratio of enzyme:protein = 1:50 (w:w), mix well, and react at 37 ˚C for 6 hours. After the reaction is completed, add 0.5 μL of formic acid (FA) to the enzymatic hydrolysate to terminate the enzymatic reaction. Centrifuge and take the supernatant for analysis. Separate the digested peptides using a C18 chromatographic column, and detect the digested peptides using a high-resolution liquid chromatograph.

S4

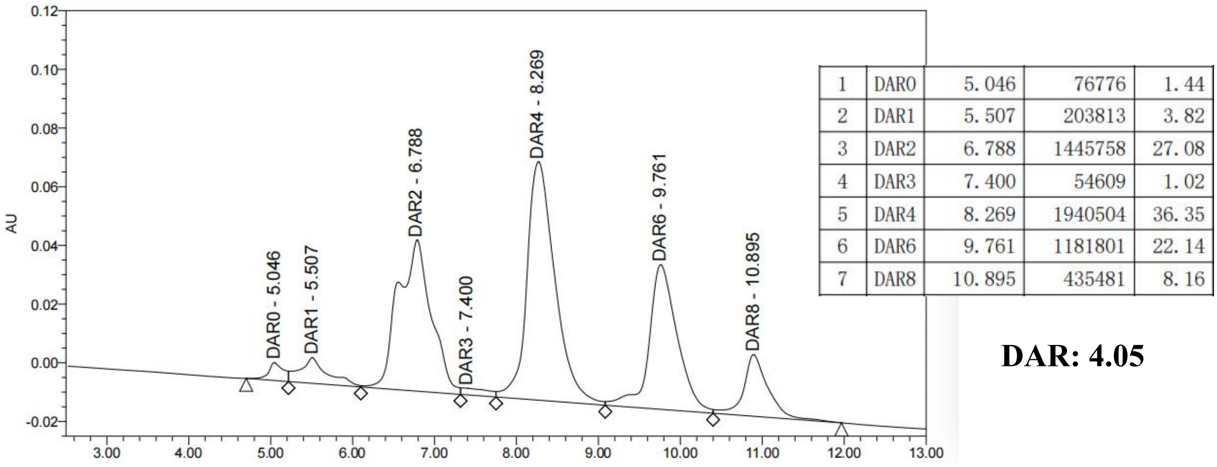

1	DAR0	5.046	76776	1.44
2	DAR1	5.507	203813	3.82
3	DAR2	6.788	1445758	27.08
4	DAR3	7.400	54609	1.02
5	DAR4	8.269	1940504	36.35
6	DAR6	9.761	1181801	22.14
7	DAR8	10.895	435481	8.16

DAR: 4.05

Mu-Nectin4-VcMMAE

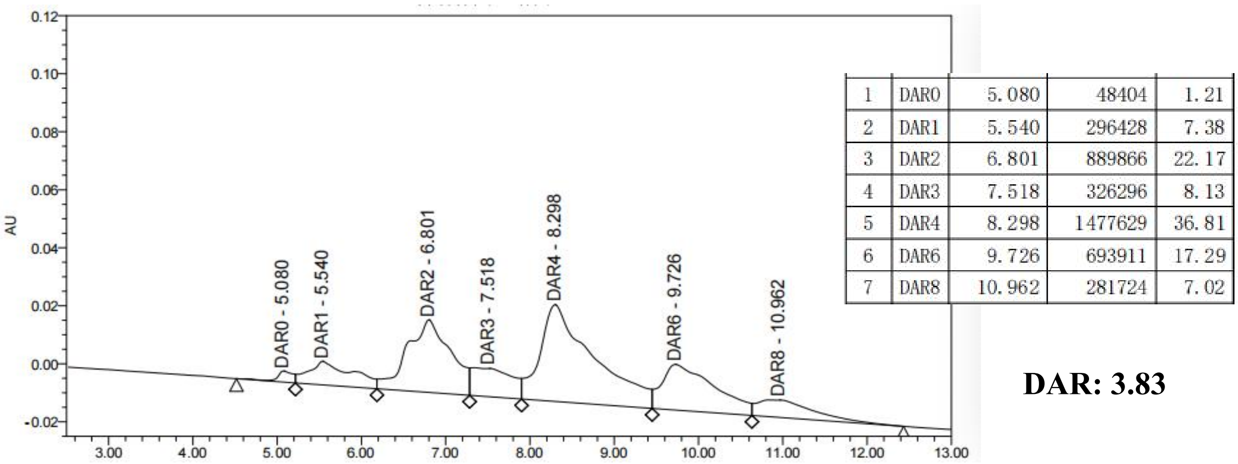

1	DAR0	5.080	48404	1.21
2	DAR1	5.540	296428	7.38
3	DAR2	6.801	889866	22.17
4	DAR3	7.518	326296	8.13
5	DAR4	8.298	1477629	36.81
6	DAR6	9.726	693911	17.29
7	DAR8	10.962	281724	7.02

DAR: 3.83

Nectin4-VcMMAE-CY3

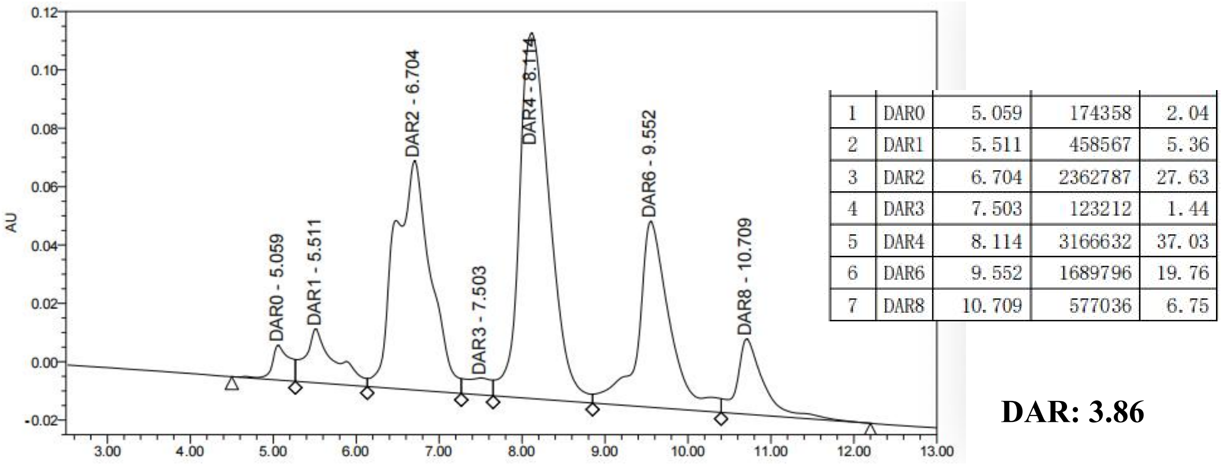

1	DAR0	5.059	174358	2.04
2	DAR1	5.511	458567	5.36
3	DAR2	6.704	2362787	27.63
4	DAR3	7.503	123212	1.44
5	DAR4	8.114	3166632	37.03
6	DAR6	9.552	1689796	19.76
7	DAR8	10.709	577036	6.75

DAR: 3.86

Mu-Nectin4-VcMMAE-CY3

(S4) Each chromatogram shows the separation results of different antibody-drug conjugates by Hydrophobic Interaction Chromatography (HIC), and the peaks are labeled with corresponding DAR values. Through calculation, it can be obtained that the DAR values of the three ADCs are all around 4, which is consistent with that of the original research drug Nectin4-VcMMAE.

HIC Method

Reagents/Materials:

Formulation buffer (10 mM sodium succinate, 120 mM sucrose, 0.12% (w/v) PS20, pH 5.3): Weigh 1.181 g of succinic acid and 41.08 g of sucrose into a beaker. Add 13.575 mL of 1 mol/L sodium hydroxide and 12.0 mL of 10% (w/v) polysorbate 20 solution. Confirm the pH is 5.3 ± 0.1, then dilute to 1 L and filter through a ≤ 0.45 μm filter.

Buffer A (1 M sodium phosphate solution): Weigh 138.0 g of NaH_2PO_4 H_2O, dissolve in 800 mL of HPLC-grade water, dilute to 1000 mL and filter through a ≤ 0.45 μm nylon filter.

Buffer B (25 mM sodium phosphate solution): Measure 25 mL of Buffer A, dilute to 1000 mL with HPLC-grade water and filter through a ≤ 0.45 μm nylon filter.

Mobile phase A (1.5 M $(NH_4)_2SO_4$ in 25 mM sodium phosphate solution, pH 6.95): Weigh 198.2 g of $(NH_4)_2SO_4$ and dissolve in 750 mL of HPLC-grade water in a beaker with stirring. Measure 25 mL of Buffer A, add HPLC-grade water to about 950 mL, mix well, adjust the pH to 6.95 with 1 M NaOH, dilute to 1000 mL with ultrapure water and filter through a ≤ 0.45 μm nylon filter.

Mobile phase B (25% isopropanol with 25 mM sodium phosphate, pH 6.95): Measure 250 mL of isopropanol and transfer to a 1 L beaker. Add Buffer B (25 mM sodium phosphate) to about 950 mL, mix well, adjust the pH to 6.95 with 1 M NaOH, add Buffer B to 1000 mL and filter through a ≤ 0.45 μm nylon filter.

Instruments: High-performance liquid chromatography equipped with an ultraviolet detector.

Test Procedure: Set the injection volume to 5 μL; Detection wavelength: 280 nm

Result Calculation:

Calculate the average DAR = (0 × % peak area0-drug) + (1 × % peak area1-drug) + (2 × % peak area2-drug) + (3 × % peak area3-drug) + (4 × % peak area4-drug) + (6 × % peak area6-drug) + (8 × % peak area8-drug) / 100

S5

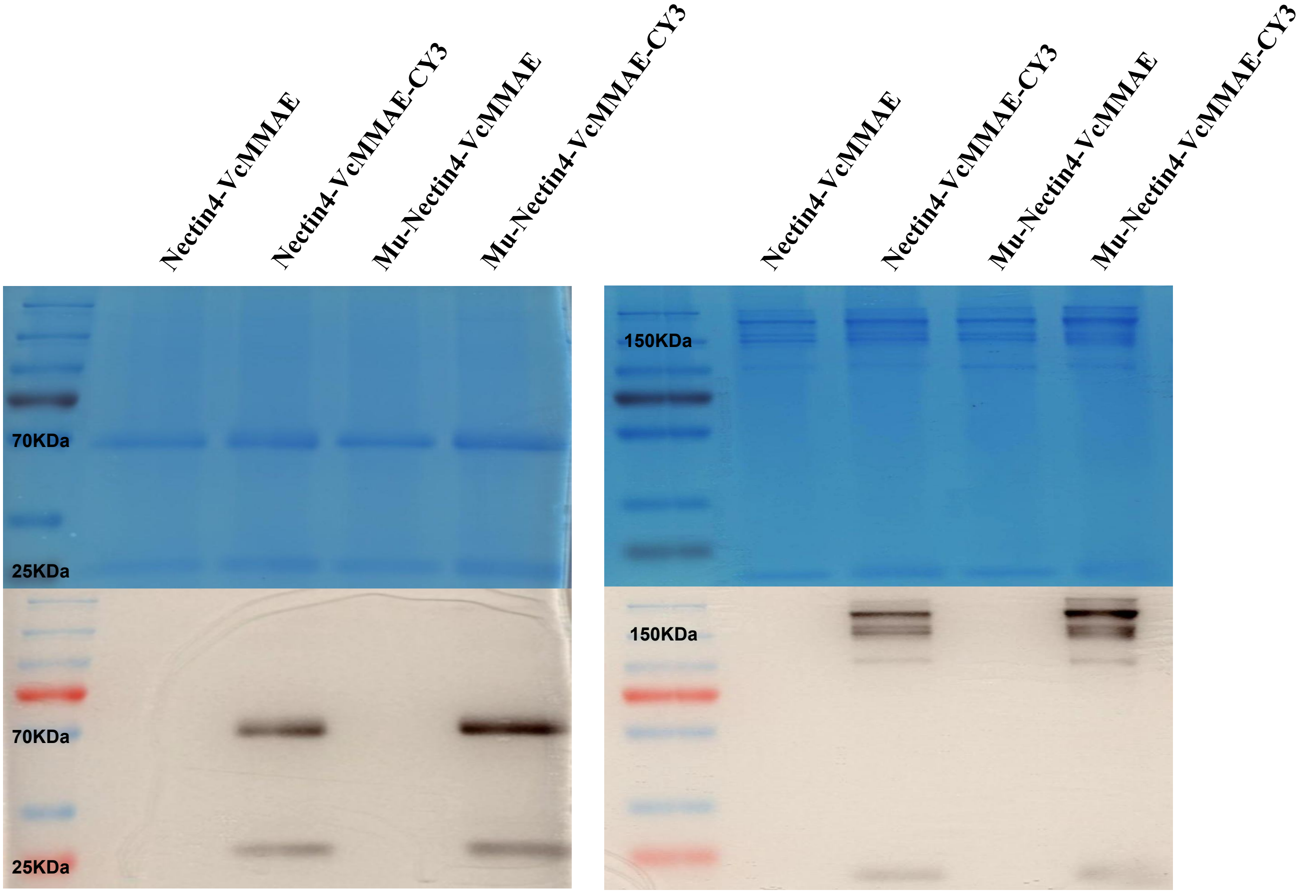

(S5) SDS-PAGE analysis under reducing conditions (Left) and non-reducing conditions (Right). SDS-PAGE analysis, followed by Coomassie Brilliant Blue staining (Up) and in-gel fluorescence imaging (Down), confirmed the successful conjugation of CY3 to the ADCs, as indicated by the co-localization of protein bands in both staining modalities.

S6

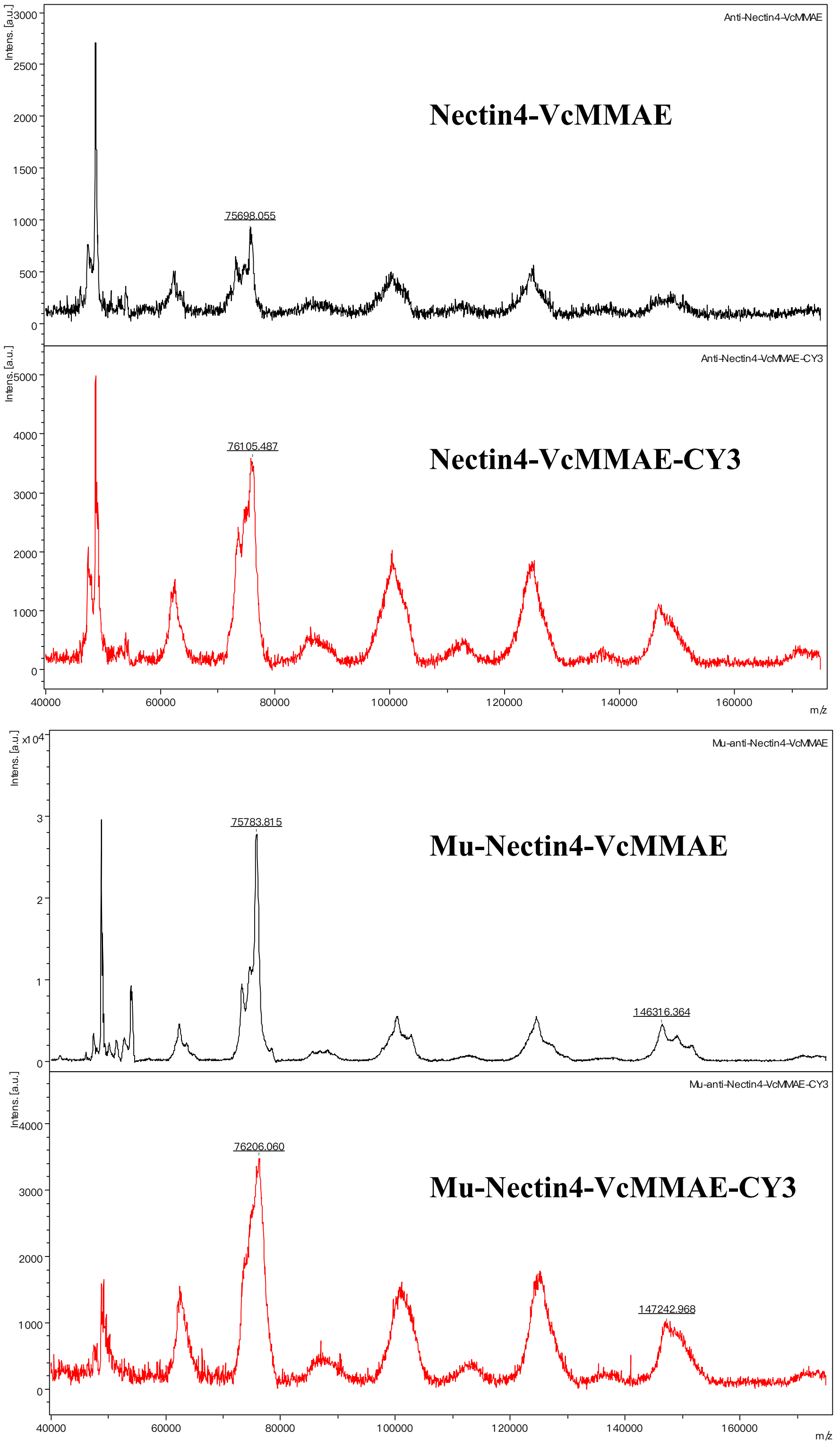

(S6) MALDI-TOF/TOF mass spectrometry analysis of CY3-conjugated ADCs. MALDI-TOF/TOF mass spectrometric analysis revealed a similar molecular weight increase in both Anti-Nectin4-Vc-MMAE and Mu-Anti-Nectin4-Vc-MMAE after CY3 conjugation, indicating that a comparable number of CY3 molecules were attached to each antibody.

Determination of Molecular Weight by Mass Spectrometry: Take 100 μg each of the reference substance and the test substance. Add 8 M guanidine hydrochloride to make the final concentration 6M. Add 1 M dithiothreitol (DTT) to make the final concentration 20 mM. Mix well, and let it react at 56° C for 30 minutes. Dilute it with ultrapure water to a concentration of 0.5 mg/mL, and then carry out MALDI-TOF/TOF mass spectrometry analysis.
